# Correlation Between Matrix Metalloproteinases With Coronary Artery Lesion Caused by Kawasaki Disease

**DOI:** 10.3389/fped.2022.802217

**Published:** 2022-02-11

**Authors:** Fang Tian, Linxi Ma, Renbing Zhao, Lijuan Ji, Xiufen Wang, Wenli Sun, Yu Jiang

**Affiliations:** ^1^Heart Center of Women and Children Hospital of Qingdao University, Qingdao, China; ^2^Department of Pediatric, Maternal and Child Health Hospital, Zibo, China; ^3^Nanchang University Queen Mary School, Nanchang, China

**Keywords:** Kawasaki disease, coronary artery lesions, matrix metalloproteinases, IVIG therapy, KD patients

## Abstract

This study was designed to clarify the role of matrix metalloproteinases (MMPs) in coronary artery lesions (CAL). Serum samples were acquired from healthy, febrile, and Kawasaki disease (KD) children with or without CAL. Standard blood parameters were examined and enzyme-linked immunosorbent assay (ELISA) was used to assess the levels of MMP-2 and MMP-9. Intravenous immunoglobulin (IVIG) therapy was conducted on the KD patients and the changes of MMPs before and after treatment were compared. The correlations between MMP levels and clinical parameters were also evaluated. Compared to febrile and healthy controls, KD patients demonstrated clinical signs characteristic of abnormal immunoregulation. However, the clinical parameters of KD patients with or without CAL were not significantly different. MMP-2 and MMP-9 levels, however, were significantly higher in KD patients with CAL than those without CAL. IVIG treatment effectively downregulated the levels of MMPs in KD patients, which was more prominent in those with CAL. Significant correlations were found between MMP levels and some clinical parameters of KD, such as fever time, white blood cell count, etc. The upregulation of MMPs significantly correlates with coronary artery aneurysms (CAAs) in KD patients, making it important biomarkers of CAL in KD patients.

## Introduction

Kawasaki disease (KD), also known as cutaneous mucosal lymph node syndrome, is a systemic vasculitis syndrome caused by an autoimmune disorder (1). KD is characterized by activation of the immune system and extensive damage to the endothelial system (2). During the acute phase of KD, the pronounced activation of immune system results in production of inflammatory mediators and proteases, as well as reactive oxygen species, which are believed to induce lesions in the vascular systems (3). Infants and young children with KD are extremely vulnerable to coronary artery lesions (CALs), including myocardial infarctions, coronary artery fistula formations, coronary artery dilatations, and coronary artery aneurysms (CAAs). Despite progresses in clinical management of KD, unfortunately 20% of children with KD progress to CAL and have a high mortality rate (4).

Matrix metalloproteinases (MMPs) are a family of enzymes responsible for degradation of extracellular matrix (ECM). The link of overexpression of specific MMPs and a number of human malignancies have been established (5). For example, the high expression of MMPs are found to be correlated with periodontal disease (6), atherosclerosis (7), cancer (8) and rheumatoid arthritis (9). MMPs are important molecules in regulating the structure and function of the coronary artery wall, therefore making them essential biomarkers and therapeutic targets for the treatment of vascular diseases (10). MMP-9 and MMP-7 have been reported to be involved in CAA (11). The pro-inflammatory factor TNF-α can induce the production of MMP-9 (12), which consequently contributes to the progression of vascular injury in KD. However, not all MMPs are effective diagnostic markers and treatment targets for CAL caused by KD and further evidences are in need to support the use of certain MMPs for accurate diagnosing and treating CAL caused by KD. Previously, MMP-2 and MMP-9 have been the focus of earlier KD research (13, 14), despite that the association of these two MMPs with CAL in KD remains unclear. In this study, we strived to analyze the relationship between MMP-2/MMP-9 and CALs in KD. By measuring serum levels of different MMPs, including MMP-2 and MMP-9, we aim to identify correlations between MMP expression and traditional clinical parameters of KD. Our findings could potentially clarify the role of MMP-2 and MMP-9 in KD, particularly those with CAL, and facilitate the development of new and effective treatment for KD.

## Methods

### Patients

The study was conducted in accordance with the Declaration of Helsinki (as revised in 2013). The study was approved by the ethics committee of Women and Children's Hospital of Qingdao University (2010071). Informed consent was taken from all the patients' parents. KD children who were admitted at Women and Children's Hospital of Qingdao University from December 2010 to July 2019 were included in the study. Diagnostic of KD was confirmed according to the criteria by the Japan Kawasaki Disease Research Committee. Exclusion criteria included: no hormones, immunosuppressants, and gamma globulin have been used in the past 2 weeks; cardiopulmonary and basic diseases; incomplete clinical data, or history of hospital transfer during treatment; unwilling to cooperate with the treatment.

### Diagnosis for Coronary Artery Dilation

The following criteria were used for diagnosis of coronary artery lesions (1): normal: Z value < 2; (2) only dilation: Z value 2 to <2.5; or initial Z value <2, Z value decline during follow-up (usually 6–12 months) ≥1; (3) small Coronary aneurysm: Z value ≥ 2.5 ~ <5; (4) Medium-sized coronary aneurysm: Z value ≥ 5 ~ <10, and absolute value of inner diameter < 8 mm; (5) Giant coronary aneurysm: Z value ≥ 10, or inner diameter. Patients with normal coronary artery (CAL-) were defined as those with Z-score < 2.0, while those with Z-score ≥ 2.0 were considered to be CAL+ (15).

### Specimen Acquisition

On the 2nd and 10th day after admission, 4 ml of peripheral venous blood was withdrawn from 140 cases of KD children after early morning fasting. Blood sample was centrifuged at 3,000 r/min for 30 min. The supernatant was collected and placed in PC Store in the tube at −75°C. Blood sample from thirty healthy children in the normal control group were blood drawn, centrifuged, and frozen in the same method.

### Statistical Analysis

Data sorting, screening and statistical analysis were performed with SPSS18.0 software: Differences between groups were compared using the Mann-Whitney U test, and subgroup analysis was performed using the Wilcoxon test. One-way ANOVA followed by Tukey's tests was applied for multiple-group comparison. Correlations analysis between MMP-2 and MMP-9 levels and clinical parameters in KD patients were performed using Pearson's correlation analysis. *P* < 0.05 was considered statistically significant.

## Results

### Study Enrollment and Cohort Characteristics

One hundred five patients with KD were initially enrolled, and eventually 95 subjects completed the study and were included for analyses. Inpatients in the Heart Center of Qingdao Women and Children hospital were analyzed for demographic and clinical parameters (Table 1). The ages of patients with KD, febrile controls (FC) and healthy controls (NC) in this study were 2.39 ± 1.65, 2.64 ± 1.97, and 2.27 ± 2.03 years old, respectively (*p* = 0.307). All groups enrolled slightly more females than males (male/female: 50/55, 25/28, and 33/39, *p* = 0.205). Compared to the healthy control group, children in the KD group and FC group demonstrated significantly longer fever time, and higher white blood cell count (WBC), red blood cell count (RBC), hemoglobin, platelet, procalcitonin (PCT), c-reactive protein (CRP), erythrocyte sedimentation rate (ESR), interleukin-6 (IL-6) and monocyte chemoattractant protein-1 (MCP-1) levels, which are signs of abnormal immunoregulation and consequence of irregular temperature regulation. These levels were even higher in the KD group than those in the FC group.

**Table 1:** Cohort characteristics.

	**KD (*n* = 95)**	**FC (*n* = 53)**	**NC (*n* = 62)**	***p*-value**
Age (y)	2.37 ± 1.54	2.64 ± 1.97	2.27 ± 2.03	0.307
Sex (male/female)	43/52	25/28	33/39	0.205
Fever time (day)	6.32 ± 1.81^***^^&&^	2.28 ± 2.55^**^	0.00 ± 0.00	<0.001
WBC (10^3^/μL)	16.43 ± 5.66^***^^&&^	10.65 ± 4.49^*^	8.54 ± 2.73	<0.001
RBC (106/mm^3^)	3.96 ± 0.47^**^^&&^	4.37 ± 0.46^*^	4.88 ± 0.61	<0.001
Hemoglobin (g/dL)	9.97 ± 1.21^*^^&^	11.15 ± 1.31^*^	11.71 ± 1.08	<0.001
Platelet (10^3^/μL)	410.64 ± 149.38^***^^&&^	354.61 ± 112.63^**^	316.45 ± 107.82	<0.001
PCT (ng/L)	1.23 ± 0.94^***^^&&&^	0.67 ± 0.26^**^	0.43 ± 0.24	<0.001
CRP (mg/dL)	58.31 ± 38.95^***^^&&&^	25.36 ± 16.84^**^	9.12 ± 6.97	<0.001
ESR (mm/h)	68.85 ± 35.07^***^^&&&^	35.88 ± 22.45^***^	12.25 ± 5.37	<0.001
IL-6 (pg/ml)	211.21 ± 30.23^***^^&&&^	105.32 ± 20.18^***^	22.19 ± 7.34	<0.001
MCP-1 (pg/ml)	204.47 ± 69.32^***^^&&^	145.37 ± 48.12^**^	104.26 ± 39.47	<0.001

*KD, Kawasaki disease; FC, febrile controls; NC, healthy controls; WBC, white blood cell counts; RBC, red blood cell counts; CRP, C-reactive protein; ESR, erythrocyte sedimentation rate; PCT, procalcitonin; IL-6, interleukin 6; MCP-1, monocyte chemoattractant protein-1*.

* *p-value of <0.05,*

** *p-value of <0.01,*

*** *p-value of <0.001 vs. NC*.

& *p-value of <0.05,*

&& *p-value of <0.01,*

&&& *p-value of <0.001 vs. FC*.

### Clinical Parameters of KD Patients With or Without CAL

We next performed analysis of clinical parameters within the KD group and strive to identify differences between those with CAL and those without CAL. We found that fever time, WBC, RBC, hemoglobin, platelet and PCT levels were not significantly different between the two groups but the levels of CRP, ESR, IL-6 and MCP-1 of those with CAL were markedly higher than those without CAL (*p* = 0.017, 0.037, 0.007, and 0.012, respectively, Table 2).

**Table 2:** Clinical parameters in two groups of KD patients.

	**KD with CAL**	**KD without CAL**	***p*-value**
	**(*n* = 50)**	**(*n* = 45)**	
Age (y)	2.58 ± 1.66	2.49 ± 1.47	0.262
Sex (male/female)	24/26	16 ± 29	0.078
Fever time (day)	6.57 ± 2.05	6.43 ± 1.68	0.446
WBC (10^3^/μL)	16.07 ± 6.79	15.32 ± 5.95	0.587
RBC (106/mm^3^)	4.05 ± 0.67	4.06 ± 0.53	0.372
Hemoglobin (g/dL)	9.93 ± 1.44	10.07 ± 1.52	0.556
Platelet (10^3^/μL)	410.43 ± 147.83	405.72 ± 155.61	0.401
PCT (ng/L)	1.27 ± 0.84	1.24 ± 0.85	0.207
CRP (mg/dL)	69.32 ± 37.04	47.53 ± 25.26	0.017^*^
ESR (mm/h)	69.74 ± 33.82	57.23 ± 34.03	0.037^*^
IL-6 (pg/ml)	210.36 ± 32.51	187.45 ± 29.48	0.007^**^
MCP-1 (pg/ml)	226.58 ± 67.35	197.21 ± 54.71	0.012^*^

*KD, Kawasaki disease; CALs, coronary artery lesions; WBC, white blood cell counts; RBC, red blood cell counts; CRP, C-reactive protein; ESR, erythrocyte sedimentation rate; PCT, procalcitonin; IL-6, interleukin 6; MCP-1, monocyte chemoattractant protein-1*.

* *p-value of <0.05,*

** *p-value of <0.01, comparison between two KD groups*.

### Analysis of MMP-2, MMP-9 Levels

We next analyzed MMP levels of patients in different groups. As shown in Table 3, MMP-2 and MMP-9 all showed markedly higher expression compared to FC and NC group (*p* < 0.001). In subgroup analysis, among KD patients, those with CAL also demonstrated pronouncedly higher MMP-2 and MMP-9 levels than those without CAL (*p* = 0.014 and 0.006, respectively). Therefore, this data implicates that MMPs likely contribute to the pathogenesis of KD and CAL.

**Table 3:** Comparison of MMP-2 and MMP-9 levels among different groups.

	**Primary group**			***p*-value**	**Sub group**		***p*-value**
	**KD**	**FC**	**NC**		**CAL**	**Non-CAL**	
MMP-2 (pg/ml)	765.47 ± 346.74^*^^&^	295.65 ± 56,32	289.42 ± 40.07	<0.001	776.43 ± 359.27	735.64 ± 321.23	0.014
MMP-9 (pg/ml)	421.94 ± 146.38^*^^&^	101.35 ± 79.43	111.79 ± 73.56	<0.001	458.94 ± 170.31	410.65 ± 197.24	0.006

*MMP-2, 9, metalloproteinases-2, 9; KD, Kawasaki disease; FC, febrile controls; NC, healthy controls; CALs, coronary artery lesions*.

* *p-value of <0.001 vs. NC*.

& *p-value of <0.001 vs. FC*.

### Reduction of MMPs by IVIG Therapy

We further analyzed the change of MMPs before and after IVIG therapy. Our data showed that after IVIG treatment, MMP-2 and MMP-9 levels of KD patients decreased 409.32 and 205.43 pg/mL, respectively, compared to pretreatment levels (*p* < 0.001 for MMP-2 and MMP-9, Table 4). Further, our analyses showed that KD patients with CAL exhibited higher reduction of MMP-2 and MMP-9 compared to those without CAL (*p* = 0.037 for MMP-2 and *p* = 0.012 for MMP-9).

**Table 4:** Changes of MMP-2, and MMP-9 levels before and after treatment.

	**KD**	**Sub group**		***p*-value**
		**KD-CAL**	**KD-NonCAL**	
ΔMMP-2 (pg/ml)	409.32 ± 213.43^***^	412.84 ± 197.48^***^	404.25 ± 204.19^***^	0.037
ΔMMP-9 (pg/ml)	05.43 ± 135.62^***^	210.77 ± 117.64^***^	186.37 ± 128.02^***^	0.012

*MMP-2, 9, metalloproteinases-2, 9; KD, Kawasaki disease; CALs, coronary artery lesions*.

*** *p-value of <0.001 pre-treatment vs. post-treatment*.

### Correlations Analysis Between MMP-2, MMP-9 Levels and Clinical Parameters in KD Patients

Next, we examined the correlation of MMP-2 and MMP-9 levels with clinical parameters of KD. As shown in Table 5, significant correlations were found between MMP-2 and MMP-9 with fever time, PCT, CRP, IL-6, and MCP-1; MMP-2 and MMP-9 also showed significant correlations with WBC.

**Table 5:** Correlations analysis between MMP-2 and MMP-9 levels and clinical parameters in KD patients.

	**MMP-2**		**MMP-9**	
	***r***	***p***	***r***	***p***
Fever time	0.307	0.015^*^	0.412	<0.001^***^
WBC	0.312	0.003^**^	0.486	<0.001^***^
RBC	0.095	0.385	0.023	0.446
Hemoglobin	−0.083	0.324	−0.091	0.179
Platelet	0.107	0.337	0.121	0.285
PCT	0.309	0.011^*^	0.397	0.003^**^
CRP	0.301	0.012^*^	0.472	<0.001^***^
ESR	0.297	0.132	0.285	0.095
IL-6	0.325	0.007^**^	0.437	<0.001^***^
MCP-1	0.298	0.037^*^	0.375	0.006^**^

*MMP-2, 9, metalloproteinases-2, 9; KD, Kawasaki disease; WBC, white blood cell counts; RBC, red blood cell counts; CRP, C-reactive protein; ESR, erythrocyte sedimentation rate; PCT, procalcitonin; IL-6, interleukin 6; MCP-1, monocyte chemoattractant protein-1*.

* *p-value of <0.05,*

** *p-value of <0.01,*

*** *p-value of <0.001*.

## Discussion

Here we report that the increased expression of MMP-2 and MMP-9 are correlated KD, particularly KD patients with CAL. The serum samples of KD patients, along with healthy patients and febrile patients as controls, were used for analysis. This approach is also adopted by another study by Takeshita et al., which showed the elevated serum levels of MMPs in KD patients. To use serum level of MMPs as biomarkers, our hypothesis was that KD is characterized by activated monocytes and neutrophils in circulation, which produce a large amount of soluble MMPs in blood. This hypothesis was adopted and tested by Korematsu et al., which showed that MMP-9 and MMP-1 were distributed predominantly in granulocytes and platelets were significant risk factors for coronary aneurysms, and correlated with neutrophil count in plasma, and the elevated MMP-1 and MMP-9 levels (16). Our study differs from the study by Korematsu et al. in that we further explored the roles of more MMPs and explored the correlation between MMP levels and clinical parameters of KD patients. The many members of MMP family have quite distinctive structures and functions and therefore it is imperative to elucidate the key MMPs with diagnostic and therapeutic values in clinical management of KD. MMP-2 is known for its role in regulating vascular smooth muscle cell migration, arterial remodeling and many endothelial dysfunction (17). MMP-3 is capable of degrading a wide range of substrates including gelatin types I, III, IV and V, and collagen types III, IV, IX and X. Besides, MMP-3 also serves as an activator of other MMPs (18). MMP-9 is responsible for degrading the subendothelial layer mainly composed of collagen, elastin and proteoglycan, which is most closely related to vascular diseases (19). To our best knowledge, most related studies supported the MMP-2 (14) and MMP-9 (20, 21) upregulation as a hallmark of KD. Consistent with the retrospective study by Wang et al. (19), which evaluated the predictive role of MMP-9 in evaluating CAL risk in KD patients, a prospective study like ours would be useful as another confirmation to facilitate the translation of the MMP-9 test in clinics. Sakata et al. reported that only MMP-9 was shown to be upregulated in KD but not MMP-2 (22). We believe this discrepancy could be attributed to the difference in subjects in their study and ours, i.e., more than half of our KD patients had CAL while Sakata et al. included subjects with no coronary sequelae. The subjects in the study by Sakata et al. also received intravenous immunoglobulin therapy, while patients who received treatments were excluded in our study. The finding that MMP-2 and MMP-9 are significantly upregulated in KD qualified the use of those three MMPs as potential biomarkers for diagnosis of KD. Further, the reduction of these MMPs by IVIG treatment justified the measurement of these MMPs in a blood test as strong indicators of therapeutic responses of KD treatment. Our results also suggest that MMP-2 and MMP-2 levels may be used to construct a mathematical model to predict CAL occurrence in KD patients, which is worth pursuing in the future.

To stratify the correlation of upregulation of MMPs with CAL caused by KD, we performed subgroup analysis, which showed that a more prominent upregulation of MMP-2 and MMP-9 was seen in KD patients with CAL, compared to those without CAL. This data indicates the role of MMP-2 and MMP-9 in the pathogenesis of CAL caused by KD, and the value of these MMPs in managing acute KD.

Prior studies have explored the role of MMP upregulation with vascular system abnormalities (5, 23). However, due to the complex role of MMPs (24), it is imperative to clarify which MMPs can serve as a therapeutic target of CAL caused by KD. The fact that some clinical parameters of KD are distinguishable between those with CAL and without CAL, such as fever time, WBC, hemoglobin, etc., while MMP-2 and MMP-9 are remarkably distinctive, suggests that the use of the levels of MMP-2 and MMP-9 for diagnosing KD are of greater clinical value. Previous reports have identified that MMP-2, 9 and 25 upregulation are correlated with abdominal aortic aneurysm (25–27). Which therefore makes them amenable molecules for surveillance and treatment of abdominal lesions. Our data show that the clinical potential of MMP-2 and MMP-9 can also be extended to CAL caused by KD. Inspired by the suppression of MMP expression by IVIG treatment, which led to alleviation of the symptoms of KD, new development of therapeutic strategies based on MMPs is desirable; the advances in targeted suppression of MMPs, e.g., by gene therapy (28, 29) may achieve a higher efficacy.

We found a significant correlation between the levels of MMPs with some protein markers of KD, including PCT, CRP, IL-6 and MCP-1, which are markers of dysregulated immunological responses. Previously, the link between MMPs and immunological responses has been demonstrated (30). Therefore, the role of MMP suppression by IVIG may also contribute to rebalance of the immune system.

## Conclusion

To conclude, here we analyzed the serum levels of three MMPs, i.e., MMP-2 and MMP-9, in KD patient with or without CAL. Healthy and febrile patients were used as controls. We found that the upregulation of the MMPs investigated in our study correlated well with KD and those with CAL demonstrated higher levels of MMPs than those without CAL, suggesting a strong link between MMPs upregulation and pathogenesis of CAL. IVIG treatment also induced a significant reduction of MMP levels. Our study indicated that MMPs are potential biomarkers of KD.

## Data Availability Statement

The raw data supporting the conclusions of this article will be made available by the authors, without undue reservation.

## Ethics Statement

The studies involving human participants were reviewed and approved by Maternal and Child Health Hospital. The patients/participants provided their written informed consent to participate in this study.

## Author Contributions

FT, LM, RZ, LJ, XW, WS, and YJ: data collection and analysis. FT and YJ: study designed and manuscript writing. All authors approved the final submission.

## Conflict of Interest

The authors declare that the research was conducted in the absence of any commercial or financial relationships that could be construed as a potential conflict of interest.

## Publisher's Note

All claims expressed in this article are solely those of the authors and do not necessarily represent those of their affiliated organizations, or those of the publisher, the editors and the reviewers. Any product that may be evaluated in this article, or claim that may be made by its manufacturer, is not guaranteed or endorsed by the publisher.
